# Sea as a color palette: the ecology and evolution of fluorescence

**DOI:** 10.1186/s40851-020-00161-9

**Published:** 2020-06-10

**Authors:** Marie-Lyne Macel, Filomena Ristoratore, Annamaria Locascio, Antonietta Spagnuolo, Paolo Sordino, Salvatore D’Aniello

**Affiliations:** Biology and Evolution of Marine Organisms, Stazione Zoologica Anton Dohrn Napoli, Villa Comunale, 80121 Naples, Italy

**Keywords:** Fluorescence, fluorescent proteins, Tree of life, Function, Metazoan, Evolution

## Abstract

Fluorescence and luminescence are widespread optical phenomena exhibited by organisms living in terrestrial and aquatic environments. While many underlying mechanistic features have been identified and characterized at the molecular and cellular levels, much less is known about the ecology and evolution of these forms of bioluminescence. In this review, we summarize recent findings in the evolutionary history and ecological functions of fluorescent proteins (FP) and pigments. Evidence for green fluorescent protein (GFP) orthologs in cephalochordates and non-GFP fluorescent proteins in vertebrates suggests unexplored evolutionary scenarios that favor multiple independent origins of fluorescence across metazoan lineages. Several context-dependent behavioral and physiological roles have been attributed to fluorescent proteins, ranging from communication and predation to UV protection. However, rigorous functional and mechanistic studies are needed to shed light on the ecological functions and control mechanisms of fluorescence.

## Background

The emission of light by living organisms relies on two primary mechanisms; natural luminescence, based on endogenous chemical reactions, and fluorescence, in which absorbed light is converted into a longer wavelength. The first observations of luminescence were made almost a century ago, when several species of hydromedusae—e.g., *Aequorea forskalea*, *Mitrocoma cellularia*, *Phialidium gregarium*, *Stomatoca atra*, and *Sarsia rosaria*—were illuminated with UV light [1, 2]. Later studies of the luminescent properties of the hydrozoan medusa *Aequorea victoria* led to the isolation of aequorin, a chemiluminescent protein that emits blue light (reviewed in [3]). The green fluorescent protein (GFP) was identified as a by-product of aequorin, and was shown to release fluorescent photons after absorbing electromagnetic energy [4] (see glossary in Table 1). The discoverers of GFP showed that calcium ion binding triggers the emission of blue light from aequorin at 470 nm, in turn prompting an energy transfer to GFP, which emits light at a longer wavelength, giving off green fluorescence at 508 nm [3, 4].

**Table 1:** Glossary

Term	Definition
Aequorin	Calcium-activated photoprotein complex responsible for luminescence in the jellyfish *Aequorea victoria*
Carotenoids	Yellow, orange, and red organic pigments produced by plants, algae, bacteria and fungi
Chlorophyll	Green organic pigment present in plants and in cyanobacteria, which is responsible for light absorption during photosynthesis and dissipates its energy by emission as fluorescence radiation
Chromatophore	Pigment-containing cell in the superficial skin tissue layer of an animal
Exitance	Totality of light leaving the surface expressed in energy or photon flux units
Fluorescence	Emission of light at a longer wavelength, in other words it is the absorption of shorter-wavelength light (excitation) followed by the release of a part of the absorbed energy at a longer wavelength (emission)
Fluorophore/ Chromophore	Part of a molecule or chemical group composed of an atom or a group of atoms responsible for the color emitted by a fluorescent protein
Green fluorescent protein (GFP)	Protein able to emit green fluorescence in the presence of short-wavelength light discovered in *Aequorea victoria*
Light absorption	Phenomenon occurring when a ray of light strikes a surface. The energy from the light (photons) is transferred to the surface material
Light scattering	Phenomenon occurring when a ray of light strikes a surface and changes its direction
Luminescence	Light generated by an enzymatic reaction (luciferase) within a living organism
Phosphorescence	Type of photoluminescence related to fluorescence displaying gradual light emission over a long period of time
Photophore	Gland or organ specialized in the production of luminescent light
Photoactivatable fluorescent proteins (PAFPs)	Class of FP capable of acute changes in their spectral properties upon irradiation with light of a specific wavelength and intensity
Pigment	Colored chemical substance found in animals or plants capable of changing color after reflection and absorption of certain wavelengths of visible light
Quenching Reflectance	Process of stopping a chemical or enzymatic reaction Fraction of photons reflected at each wavelength
Sandercyanin	Lipocalin family protein, isolated from a freshwater fish, able to bind to biliverdin IXα displaying blue color naturally, or red fluorescence under UV radiation
UnaG	Fatty acid binding protein (FABP), isolated from marine eels, able to bind endogenous bilirubin triggering green fluorescence

Technical terms defined to clarify concepts in the field of natural fluorescence

Green fluorescent protein consists of a single polypeptide chain of 238 amino acids in length, and does not require a cofactor [5]. The chromophore, the structural feature of GFP responsible for color emission, is formed by the autocatalytic cyclization of the tripeptide 65-SYG-67 [3]. Members of the GFP family constitute a distinct protein class, all of which share similar structures [6]. After the cloning of the GFP gene [7] and the in vivo demonstration that its recombinant expression in *Escherichia coli* and *Caenorhabditis elegans* induces fluorescence [8], interest in and applications of GFP have continued to increase within the scientific community. Originally used as a reporter gene for tracing proteins, organelles, and cells, non-invasive GFP tagging has become a routine tool in scientific research for a variety of experimental approaches, such as gene reporting, drug screening, and labeling. In parallel to the discovery of new wild-type fluorescent proteins (FPs), the hunt to engineer novel FP mutants has led to modifications of their chemical properties in an effort to broaden their potential applications in cell biology and biomedicine [9]. Beyond the biotechnological revolution prompted by the discovery of FPs, no mechanistic explanation has been proposed for the presence of fluorescence in nature. Recent findings relating to new FPs have prompted investigations in novel research directions, such as evolutionary ecology, as little is known about the eco-physiological role of fluorescence in nature. In the present review, we present and discuss several perspectives, such as the phylogenetic distribution of fluorescence in nature, the expansion of FPs in the tree of life, pigment-generated fluorescence, and the ecological functions of fluorescence in aquatic and terrestrial environments.

### Differences between marine fluorescence and luminescence

In the sea, the sources of light energy are sunlight, moonlight, and luminescence. Only a small fraction of daylight penetrates the ocean’s depths, becoming progressively dimmer before resolving to a uniform blue spectrum (470–490 nm) light. Orange-red light penetrates only to a depth of 15 m and ultraviolet light to 30 m [10]. Bioluminescence is the emission of visible light by an organism resulting from luciferin oxidation under the control of luciferase. Instead, photoproteins, which are the primary substrates of the light-emitting reactions of various bioluminescent organisms in diverse phyla, do not require luciferase enzyme activity [11], but instead rely on Ca^2+^ or superoxide radicals and O_2_ to trigger bioluminescence. This mechanism is the primary source of biogenic emission of light in the ocean from the epipelagic to the abyssal zone, in regions from the poles to the equator [12]. For many marine species, the primary visual stimulus comes from biologically generated light rather than from sunlight. Given its widespread distribution, bioluminescence is clearly a predominant form of communication in the sea, with important effects on diurnal vertical migration, predator–prey interactions, and the flow of material through the food web [12].

**Fig. 1:**
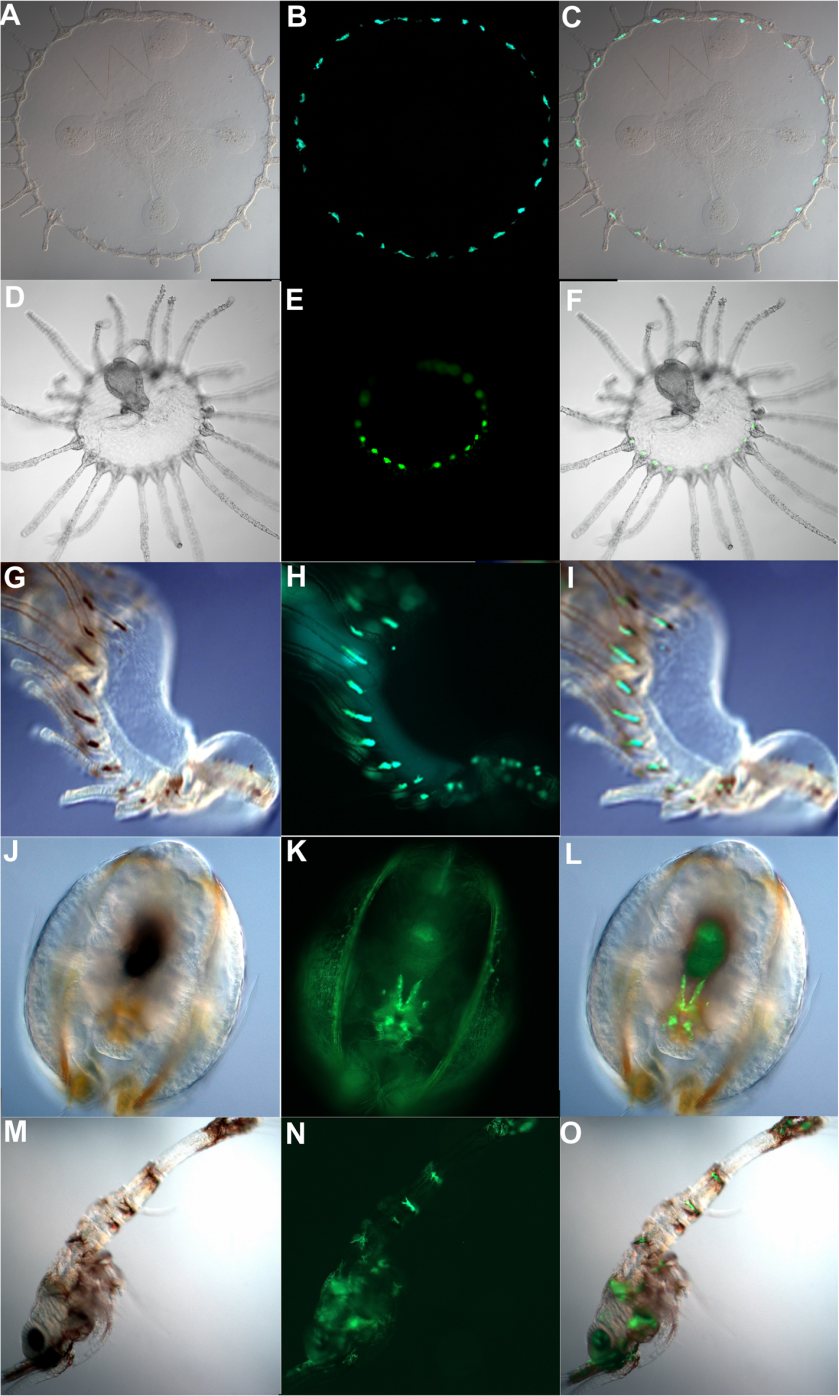
Samples of GFP in cnidarians and pigment generated by fluorescent organisms from the Gulf of Naples (Italy). **a–f**: Cnidarian hydrozoans, *Clytia hemisphaerica***a–c** and *Obelia* sp. **d–f**; **g–i**: Phoronida, actinotroch larva of unknown species; **j–o**: Arthropoda, unknown ostracod species **j–l** and unknown crustacean species **m-o**

Biofluorescence is a phenomenon dependent on external light, in which a fluorophore converts absorbed short-wavelength light to a longer wavelength. In other words, incident light is re-emitted at a longer, less energetic wavelength, therefore with low energy conversion efficiency. Natural fluorescence may derive not only from fluorescence-emitting proteins, but also from organic pigments, such as chlorophyll, carotenoids, flavonoids, pterins, or minerals, such as zinc, strontium, aluminium, selenium, and cadmium, that are able to emit light at similar wavelengths (Table 2 and Fig. 1) [33]. In terrestrial animals, specific compounds, such as chemical derivatives of the organic substance coumarin, seem to be at the origin of fluorescence observed in the cuticles of some arthropods (e.g., spiders and scorpions) [26, 34]. These biotic and abiotic substances may also be phosphorescent under UV light, a fluorescence-like physical process characterized by a longer emission time course. Especially in aphotic habitats, fluorescence and luminescence may coexist and interact within the same organism, in which the latter acts as light energy source for fluorescence since some luminescent compounds (e.g., those in dinoflagellates) may also be autofluorescent [35]. By virtue of this coexistence, photophores (luminescent organs) often convert their naturally blue luminescent light into green light by using GFP [13, 20]. This is also the case of chromatophores, which are dermal cells that mediate color changes in vertebrates (see glossary in Table 1). In fish, for example, these cells are specialized in the synthesis and storage of light-absorbing pigments [36].

**Table 2:** Natural and photoactivable fluorescent proteins and pigments

Gene ortholog	Protein class	Phylum/Organism	λExc (nm)	λEmi (nm)	Reference
GFP	GFP	Cnidaria, Hydrozoa	395	510	[13]
DsRed	GFP	Cnidaria, Anthozoa	558	583	[14]
cFP484	GFP	Cnidaria, Anthozoa	456	484	[14]
zFP538	GFP	Cnidaria, Anthozoa	528	538	[14]
ZsGreen	GFP	Cnidaria, Anthozoa	496	506	[14]
asulCP	GFP	Cnidaria, Anthozoa	572	595	[15]
cgigGFP	GFP	Cnidaria, Anthozoa	399	496	[15]
hcriGFP	GFP	Cnidaria, Anthozoa	405	500	[15]
dis3GFP	GFP	Cnidaria, Anthozoa	503	512	[15]
dendFP	GFP	Cnidaria, Anthozoa	492–557	508–575	[15]
mcavGFP	GFP	Cnidaria, Anthozoa	506	516	[15]
mcavRFP	GFP	Cnidaria, Anthozoa	508	580	[15]
rfloGFP	GFP	Cnidaria, Anthozoa	508	518	[15]
rfloRFP	GFP	Cnidaria, Anthozoa	566	574	[15]
scubGFP1	GFP	Cnidaria, Anthozoa	497	506	[15]
zoan2RFP	GFP	Cnidaria, Anthozoa	552	576	[15]
asCP562	GFP	Cnidaria, Anthozoa	562	595	[16]
Kaede	PAFP	Cnidaria, Anthozoa	508–572	518–580	[17]
Dendra	PAFP	Cnidaria, Anthozoa	488–556	505–575	[18]
Dronpa	PAFP	Cnidaria, Anthozoa	503	518	[19]
pmeaGFP1	GFP	Arthropoda, Copepoda	489	504	[20]
GFPa1	GFP	Chordata, Cephalochordata	497	516	[21]
UnaG	Fatty Acid binding	Vertebrata, Teleostea	500	527	[22]
Sandercyanin	Lipocalin	Vertebrata, Teleostea	375	630	[23]
SmURFP	Phycobiliprotein	Cyanobacteria	642	670	[24]
**Pigment name**	**Pigment type**	**Phylum/Organism**	**λ**_**Exc**_**(nm)**	**λ**_**Emi**_**(nm)**	**Reference**
Crustacyanin	Carotenoid	Arthropoda Malacostraca	530	580	[25]
β-carboline	Tryptophan derivative	Arthropoda Aracnida	360–370	445–490	[26]
Psittacofulvin	Non-carotenoid	Chordata Aves	N/A	N/A	[27]
Spheniscins	Pterins-like	Chordata Aves	UV	N/A	[28]
Sepiapterin	Pteridin	Chordata Actinopterygii	UV	450–490	[29]
Porphyrin	Porphyrin	Mollusca Gastropod	UV	625	[30]
Hyloin	Dihydroisoquinolinone	Chordata Amphibia	390–430	450–470	[31]
Betaxanthins	Betalains	Plantae caryophyllales	463–474	509–512	[32]

Fluorescent molecules, their taxonomic distribution, excitation/emission wavelengths, and the original scientific reference. Fluorescent proteins belong to three different classes: GFPs, fatty acid binding proteins, and lipocalins. Various pigments can also participate in the process of fluorescence

### Fluorescent proteins in the tree of life

The evolutionary origin of FP genes in metazoans remains subject to debate. Canonical GFP orthologs have been identified only in the phyla Cnidaria, Arthropoda, and Chordata, suggesting the presence of GFP in the last common ancestor of all metazoans (Fig. 1). Recently, GFP-like genes have been found in transcriptomes of 30 ctenophores, which is relevant to their early divergent phylogenetic position [37]. Although one of them was initially described as a fluorescent protein [38], a deeper study indicated that fluorescence in the ctenophore was not intrinsic, but originated from a siphonophore it had consumed [37].

**Fig. 2:**
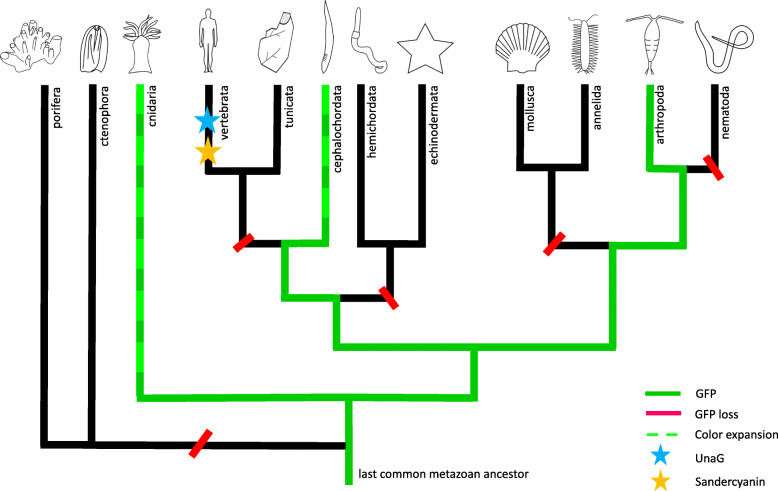
Distribution of fluorescent proteins in metazoan. Canonical GFPs have been found in cnidarian, arthropods and cephalochordates, supporting the hypothesis of a common metazoan ancestral origin. Color expansion is present in cnidarians and has been recently showed in cephalochordates as well. Furthermore, two other FPs, not related to GFP, have been characterized in vertebrates: UnaG and Sandercyanin

For the present review, we performed an extensive search for well-annotated transcriptomes and genomes of sponges, nematodes, annelids, molluscs, echinoderms, and hemichordates, but did not identify any orthologs of canonical GFPs. This suggests that independent gene loss events occurred in the evolutionary history of several animal clades. An alternative phylogenetic scenario capable of explaining the scattered phylogenetic distribution of GFPs would involve independent horizontal gene transfer events, probably through diet; this possibility requires further investigation [39]. Two important evolutionary events appear to have occurred chordate clade: the loss of GFP representatives in Olfactores (comprising tunicates and vertebrates) and, in contrast, extraordinary gene expansion recently detected in cephalochordates (Fig. 2). In fact, 21 expressed GFPs have been identified in the amphioxus *Branchiostoma lanceolatum*, although the significance of this extensive number of GFPs requires further functional clarification [6, 40].

Recently, new FP families have been characterized in vertebrates bearing different features in comparison with canonical GFP proteins. For instance, a blue fluorescent protein named Sandercyanin was first isolated almost a decade ago from the freshwater walleye (*Sander vitreus*), a fish founds in the lakes of North America (Fig. 1). This is the first FP described with blue absorption and far-red emission under UV radiation [23]. Furthermore, a non-GFP green fluorescent protein belonging to the fatty-acid-binding protein family (FABP) was isolated from the muscles of the freshwater eel *Anguilla japonica* (Fig. 2) [22]. This protein, named UnaG (*unagi* is the Japanese name for this species of eel), triggers bright green fluorescence through coupling with bilirubin [22]. Two novel brightly fluorescent FABP proteins originating from a gene duplication event have also been characterized in the false moray eel (*Kaupichthys hyoproroides*) [41]. Since this cryptic eel occupies a nearly monochromatic marine environment predominated by blue wavelengths, further analyses are needed to determine the ecological function of this green emission [41].

Finally, fluorescence has been recently identified in two catshark species, *Cephaloscyllium ventriosum* (swell shark) and *Scyliorhinus rotifer* (chain catshark), in which light emission from the skin is essentially due to bromo-kynurenin yellow metabolites. This discovery raises new questions about the diversity of fluorescence sources in nature and the ecological roles in vertebrates [42].

A wide range of color-emitting GFPs characterize the phylum Cnidaria, in particular in anthozoans (sea anemones and corals). More than two decades ago, the DsRed protein was discovered in non-bioluminescent reef coral species of the genus *Discosoma* [14]. Chromophore synthesis, responsible of the color of the protein, is a molecular process that requires genomic stability, as any mutation disrupting the autocatalytic reaction in DsRed would convert it into green protein [43]. Indeed, at least seven different mutant variants of DsRed emitting in the green range have been generated by random and site-specific mutagenesis events [44, 45].

Anthozoan FPs have been engineered to produce photoactivable FPs (PAFPs) generating huge light-induced spectral changes. Dendra, originally from octocoral *Dendronephthya* sp., was the first PAFP shown to be capable of photoconversion from green to red fluorescent states in response to either visible blue or UV-violet light [18]. In addition to its high photostability, this PAFP is easily photoactivated by ordinary 488-nm laser light. Similar to Dendra, Dronpa is a reversible bright green PAFP derived from the coral *Echinophyllia* sp. that shows interesting properties beyond its extreme brightness, as it can be switched on and off repeatedly with high contrast and a minimal loss in fluorescence intensity [19, 46].

While FPs have been characterized mainly in eukaryotes, interest in prokaryote orthologs has increased in the last years. A far-red Biliverdin-Binding FP (smURFP) was developed from a member of the same family as Sandercyanin derived from the cyanobacterium *Trichodesmium erythraeum*. Unlike Sandercyanin, smURFP fluorescence is visible without exogenous biliverdin, and is the brightest far-red/near-infrared FP created to date [24]. Bacterial fluorescence has also been a template for generating non-oxygen-dependent FPs. Flavin mononucleotide (FMN)-based fluorescent proteins (FbFPs), unlike GFPs, do not require oxygen as a cofactor to synthesize the FMN chromophore, which makes these FPs very convenient for studying anaerobic biological systems [46].

### Ecological functions of fluorescence

Compared to terrestrial animals, marine organisms occupy a spectrally limited visual environment, in which their eyes are adapted to different light conditions. In particular, a topic of interest in the evolutionary and ecology fields concerns how different types of visual systems developed specific spectral receptors and pigments (allowing them to detect fluorescence). Crustaceans such as the mantis shrimp have developed a fascinating system of color vision, based on at least eight primary spectral receptors ranging from 400 to 700 nm. In the species *Lysiosquillina glabriuscula* it has been shown that, at depths of 20, 30, and 40 m, fluorescence contributes 9, 11, and 12% of the photons that stimulate the shorter-wavelength receptor, and 15, 22, and 30% of those stimulating the longer-wavelength receptor, respectively [47].

However, to determine whether sufficient energy is transferred in order to make a meaningful difference to the visual signal under natural lighting conditions, several optical factors such as exitance and reflectance (see glossary, Table 1) need to be calculated. In addition, fluorescence can only play a role in vision if it contributes to the total light leaving the surface and to a behavioral response; i.e., if the behavior of the viewing organism is influenced by the presence or absence of fluorescence in the subject [48]. Another key unresolved issue is whether fluorescence is sufficiently bright to be visible against the background light environment.

What are the adaptive advantages conferred by fluorescence? While it is thought that not all biofluorescence is functionally relevant, few examples of its ecological role have been described. A number of hypotheses have been advanced to explain the roles of fluorescence, alone or in combination with luminescence. These include photoprotection for stem cells, photosynthesis enhancement, predation by prey lure or distraction, and protection against oxidative stress. Below, we use a taxonomic approach to review advances in the understanding of the ecological roles of fluorescence in marine organisms.

Green fluorescent proteins and, in general, fluorescent pigments, act as a photoprotective system against damage from sunlight [49]. It has been shown that UV_A_ and extreme photosynthetically active radiation (PAR) trigger photodamage and photoinhibition in coral-dinoflagellate symbiosis that, in severe cases, may result in coral bleaching [49, 50]. In this context, FPs histologically positioned above endosymbionts may function as an energy dispatcher through fluorescence and light scattering. In the hydrozoan *A. victoria*, the response to superoxide radicals was investigated by examining the protein structure of GFP. Superoxide radicals and reactive oxygen species are typically present in the hyperoxic conditions that these organisms experience during the daytime due to the photosynthetic activity of algal symbionts. It has been shown that GFP can quench (see glossary, Table 1) these superoxide radicals without altering its fluorescence properties [51], thereby providing protection from antioxidants.

### Marine organisms

It is fascinating how in the deep sea, the largest habitat on earth, marine organisms can live in constant darkness without access to high-energy blue light [52], using instead luminescence as the predominant light-signaling phenomenon. It is even more fascinating that, as a consequence of the production of blue luminescent light in this habitat, fluorescence acts as an energy-collecting device that enhances photosynthesis in cnidarians [53].

The tentacles of the deep-sea anemone *Cribrinopsis japonica* emit green fluorescence, when excited by blue light, potentially as a lure for prey attraction [54]. Interestingly, the GFP isolated from this anemone is more stable than other GFPs; however, it is unclear whether this results from adaptation to its deep-sea habitat. The sea anemone *Nematostella vectensis* was the first early metazoan whose genome was sequenced, and represents a powerful model system for evolutionary development biology [55]. This species possesses seven GFP genes, of which only *nvfp-7r*, which codes for a red fluorescent protein (NvFP-7R), is functionally fluorescent. The transcriptional regulation of the *nvfp-7r* gene shows spatiotemporal complexity as well as the unexpected capacity to respond to positional information in the adult body plan [56]. Despite the current knowledge of the functional significance of red fluorescence in *N. vectensis* (as well as in the vast majority of fluorescent organisms), it is nonetheless based upon hypothetical reconstructions. The large toolkit of sophisticated approaches available for this species renders this small anthozoan a promising model for the acquisition of deeper insights into the role(s) of fluorescence.

The function of fluorescence in prey attraction has been assessed in a non-luminescent hydromedusa species, *Olindias formosus*, which possesses fluorescent and pigmented patches on the end tips of its tentacles from early development of the polyp stage. In laboratory experiments under blue light conditions, these pigmented patches attract juvenile rockfish of the genus *Sebastes*, which do not respond in the absence of fluorescence [57]. A similar mechanism has been observed in the siphonophore *Resomia ornicephala*, which possesses fluorescent tentacles that attract and capture euphausiid shrimp [58].

In the hydrozoan jellyfish *Clytia hemisphaerica*, the intense green fluorescence observed in the endodermal and ectodermal cells of the mouth, stomach, and gonads may have several functions, including protection of stem cells and maternal mitochondrial DNA from UV light. Each of the four GFPs (and the three aequorins) isolated in this species show life-cycle stage and tissue specificity, supporting the hypothesis that fluorescence has acquired multiple specialized roles in response to environmental (depth), physiological (life-cycle) or behavioral (spawning) conditions [59]. The siphonophore *Erenna sirena* is another example in nature of energy conversion from luminescence to fluorescence by creating yellow to red fishing lures (583–680 nm) on its tentacles surrounded by a luminescent photophore [60].

In the phylum Arthropoda, few copepod species exhibit luminescence, while several others, belonging to the Pontellidae and Aetideidae families, exhibit biofluorescence, which is thought to serve as a mate perception and attraction signal and/or a camouflage mechanism [20, 61]. Interestingly, the high brightness and stability and low cytotoxicity of copepod GFP proteins make these molecules particularly well-suited to a variety of molecular and biological applications [62].

Although neither stomatopod crustaceans nor mantis shrimps possess fluorescent proteins, many species display a very bright fluorescent coloration that is used in postural signaling to increase shrimp visibility when sensing a predator, for intra-species competition with other males, and in mate choice [47].

Studies on FPs in chordates provide further information about their function when compared to invertebrates. In the cephalochordates *Branchiostoma floridae, Branchiostoma lanceolatum, Branchiostoma belcheri* and *Asymmetron lucayanum,* an expansion of GFPs has been reported in the genome [6, 21, 63]. Different functions have been postulated, such as playing a role in antioxidant mechanisms by scavenging deleterious oxy-radicals, in photoprotection and attracting motile planktonic prey [6, 21]. In cartilaginous and bony fish, such as catsharks and reef fish, fluorescence may function in communication, species recognition, and camouflage [64] or it may simply be a chemical by-product of skin composition.

Green and red fluorescence have also been observed in the sea turtles *Eretmochelys imbricata* and *Caretta caretta*. Whether these originate from diet (corals, zooxanthelles) [65], or as a by-product of the chemical composition of algae growing on their shells [66] remains unclear. It is also uncertain what role fluorescence might play in a sea turtle. In the case of the loose-jaw dragonfish *Malacosteus* sp., the animal emits luminescent light and far-red fluorescence, which may be used in predation [67]. Most reef fish possess visual pigments ranging from UV to green wavelengths [68, 69]. Interestingly, red fluorescence has been observed in more than 180 species of marine fish [52], strongly suggesting its potential role in vision [69].

Experiments conducted on the diurnal fish *Cirrhilabrus solorensis*, whose visual system is receptive to deep red fluorescent coloration, have demonstrated that its strong red fluorescence emission body pattern affects male–male interaction [70]. A study on the spectral sensitivity of the goby *Eviota atriventris* revealed that this fish possesses long-wavelength visual pigments, making it physiologically sensitive to red fluorescent coloration [71]. Lending further weight to this hypothesis, yellow intraocular filters have been found in reef fish, sharks, lizardfish, scorpionfish, and flatfish, which could enable them to detect fluorescence [72]. It has also been demonstrated that sharks and rays are capable of visualizing their own fluorescence, showing sexually dimorphic fluorescent body patterns, which is suggestive of a function in communication or species-recognition role [64].

Nevertheless, despite several attempts, no sufficient experimental studies have clarified the functional and behavioral link between fluorescence and vision in organisms with complex visual systems [69].

### Terrestrial organisms

Fluorescence produced by fluorescent metabolic chemical by-products is also observed in many terrestrial organisms. Several recent studies have suggested ecological and behavioral roles similar to those highlighted in marine organisms. In amphibians, the tree frog *Hypsiboas punctatus* emits hyloins, fluorescent compounds secreted from the lymph and gland nodes [31]. This suggests that fluorescence is part of the integumentary pigment system in this amphibian, representing a novel extra-chromatophore source of coloration. In low-light conditions, frog fluorescence accounts for 18–29% of the total emerging light comprising fluoresced and reflected photons. This confers greater brightness to *H. punctatus* and matches the sensitivity of night vision in this clade.

Another interesting example is represented by butterfly wings, which possess an intrinsic controlled system that is remarkably similar to recent LED technology, utilizing a photon crystal-like structure capable of producing directed fluorescence [73].

Behavioral experiments performed in arthropods and chordates underline the potential role of fluorescence in communication. In fact, the fluorescent plumage in the parrot *Melopsittacus undulate* was shown to have behavioral implications in mate selection, rather than in social communication [74]. In fact, female and male parrots exhibit significant preferences for fluorescent birds of the opposite sex [75].

The jumping spider *Cosmophasis umbratica* has been shown to interact differently in the presence of UV reflectance or UV-induced fluorescence while testing sex-specific courtship signaling [76]. Males present UV-reflective patches of scales on the face and body that are shown during conspecific posturing [77]. These patches are lacking in females, which instead have palps with a UV-excited bright green fluorescence that are absent in males. During the experiment, female spiders made a postural response to male courtship under full-light spectrum, while they did not respond or turned away without UV. Similarly, males ignored non-fluorescing females. The courtship responses of the spiders were an effect of sexual coloration instead of behavioral changes. To determine this, the behavioral responses of individuals of one sex under full-spectral light were compared when the partner of the opposite sex was illuminated with UV-deficient light. Most UV-irradiated male spiders that courted fluorescent females failed to do so when the female lacked fluorescence even though her response was the same as under normal light.

Desert scorpions exhibit blue/green fluorescence under UV light in laboratory conditions, although this phenomenon does not manifest in natural daylight conditions. Beta-carboline, a tryptophan derivative molecule is responsible for the fluorescence in the cuticle of scorpions (Table 2) [26]. Although it has been hypothesized that fluorescence in scorpions may serve as a prey lure, it is clear that the formation of this substance on the cuticle of this animal serves no function [78].

Finally, it has been recently shown that tubercles protruding from the skull of chameleons reveal fluorescence upon short-wavelength UV-irradiation; this may play a role in species recognition [79]. Emission signals corresponding to deep blue are reasonably rare in tropical forests; this form of biofluorescence thus appears to be a distinct signal against the green vegetation background reflectance [79].

Fluorescence plays roles in terrestrial animals as well as plants, such as the carnivorous *Nepenthes*, *Sarracenia*, and *Dionaea*. Preliminary studies have quantitatively measured fluorescence in flowers in several species and concluded that its relevance for communication is negligible [80]. However, more recently, blue fluorescence emission at the catch sites of these plants was detected, suggesting that it could play a role in attracting arthropod prey compared to non-illuminated plants [81]. In the yellow flowers of the plant *Portulaca grandiflora*, the pigment betaxanthin is at the origin of green emission when the flower is excited by blue light (Table 2) [32]. The flower exhibits natural yellow coloration; its brightness may be increased by this fluorescent pigment at a particular wavelength, making the flower more visible to pollinators.

## Conclusion and perspectives

Both fluorescence and luminescence are prevalent optical processes present in nature and crucial for species communication and predator–prey interactions, and may coexist or cooperate in many species, such as deep-sea animals. The discovery of GFP in 1962 in the cnidarian jellyfish *A. victoria* and the subsequent characterization of numerous GFPs in several taxa have prompted research on biotechnological applications. More recently, orthologs of GFP have been identified in arthropods and chordates; nevertheless, the evolutionary and ecological significance of fluorescence requires substantial further study.

Recently, the exciting discovery of novel types of fluorescent proteins in vertebrates (i.e. UnaG and Sandercyanin) has led to novel evolutionary insights, given that until recently only GFPs and GFP-like proteins were thought to support fluorescence. The identification of yellow fluorescent metabolites in sharks has also opened new avenues of inquiry into their roles in central nervous system function, photoprotection, and resilience to microbial infections. For example, genome editing of fluorescent proteins in a living model organism such as *Clytia hemisphaerica* may be highly informative in order to assess its biological function. Although its roles in communication, predation, and camouflage in several taxa are widely accepted in the scientific community, evidence for and interpretation of additional functions require stronger scientific support. It will also be crucial to conduct further functional studies of fluorescence in both terrestrial and marine species to assess whether the emission of fluorescence is quantitatively significant in natural environments, which is necessarily different from that under laboratory conditions.

Finally, our understanding of the role of fluorescence in animal vision is in its early stages, as seen from a few recent studies in reef and deep-sea fish. Further research is also needed to clarify the anatomy of the visual apparatus, which we did not examine in this review, and the molecular toolkits involved in color, contrast, and fluorescence detection, in order to shed light on these unresolved questions.

## Data Availability

Not applicable.
